# Experimental and *in silico* evaluation of *Carthamus tinctorius* L. oil emulgel: a promising treatment for bacterial skin infections

**DOI:** 10.3389/fcimb.2023.1253095

**Published:** 2023-09-05

**Authors:** Javaria Saeed, Syed Nisar Hussain Shah, Hina Javed, Asma Aslam, Anam Ali, Farhan Siddique, Tahreem Zahra, Yousef A. Bin Jardan, Gezahign Fentahun Wondmie, Hiba-Allah Nafidi, Mohammed Bourhia

**Affiliations:** ^1^ Department of Pharmaceutics, Faculty of Pharmacy, Bahauddin Zakariya University, Multan, Pakistan; ^2^ Faculty of Pharmacy, The University of Lahore, Lahore, Pakistan; ^3^ Department of Pharmacology, Faculty of Pharmacy, Bahauddin Zakariya University, Multan, Pakistan; ^4^ Department of Pharmaceutical Chemistry, Faculty of Pharmacy, Bahauddin Zakariya University, Multan, Pakistan; ^5^ School of Pharmaceutical Science and Technology, Tianjin University, Tianjin, China; ^6^ Institute of Zoology, Bahauddin Zakariya University, Multan, Pakistan; ^7^ Department of Pharmaceutics, College of Pharmacy, King Saud University, Riyadh, Saudi Arabia; ^8^ Department of Biology, Bahir Dar University, Bahir Dar, Ethiopia; ^9^ Department of Food Science, Faculty of Agricultural and Food Sciences, Laval University, Quebec City, QC, Canada; ^10^ Department of Chemistry and Biochemistry, Faculty of Medicine and Pharmacy, Ibn Zohr University, Laayoune, Morocco

**Keywords:** *Carthamus tinctorius* L oil, extraction, anti-bacterial, anti-oxidant activity, molecular docking

## Abstract

**Purpose:**

The current study aimed to develop a topical herbal emulgel containing *Carthamus tinctorius* L. (CT) oil extract, which has been scientifically proven for its antibacterial and antioxidant activities for the ailment of bacterial skin infections.

**Method:**

The CT emulgel was formulated by response surface methodology (RSM) and was evaluated by various parameters like extrudability, spreadability, pH, viscosity, and antibacterial and antioxidant activities. Molecular docking was also performed using AutoDock.

**Results:**

Among all formulated CT emulgels, F9 and F8 were optimized. Optimized formulations had shown good spreadability and extrudability characteristics. Sample F8 had % inhibition of 42.131 ± 0.335, 56.720 ± 0.222, and 72.440 ± 0.335 at different concentrations. Sample F9 had % inhibition of 26.312 ± 0.280, 32.461 ± 0.328, and 42.762 ± 0.398 at concentrations of 250 µg/ml, 500 µg/ml, and 1,000 µg/ml, respectively, which shows that both samples F8 and F9 have significant antioxidant potential. Optimized CT emulgels F8 and F9 had significant antibacterial activity against *Staphylococcus aureus* and *Escherichia coli* at p-value = 0.00, the Emulgel-F8 shows zone of inhibition of 24 mm for *E-coli* and 19 mm for *S-aureus*. Emulgel-F9 shows zone of inhibition of 22 mm for *E-coli* and 15 mm for *S-aureus* while pure CT- Oil extract shows zone of inhibition of 25 mm for *E-coli* and 20 mm for *S-aureus* and ciprofloxacin used as standard shows 36mm zone of inhibition against both *E-coli* and *S-aureus*. The comparative investigation through molecular docking binding affinities and interactions of ligands with various target proteins provides insights into the molecular processes behind ligand binding and may have significance for drug discovery and design for the current study.

**Conclusion:**

The current study suggests that *C. tinctorius* L.-based emulgel has good antioxidant and antibacterial activities against *E. coli* for the treatment of bacterial skin infections.

## 1 Introduction

Transdermal drug delivery system (TDDS) defines the dosage form that distributes an optimum drug quantity across the skin. Its success overlaps the traditional processes including injectable and oral drug delivery (Kumar et al., 2016). It increases patient compliance, as the drug bypasses first-pass metabolism through TDDS. It also decreases the untoward effects of a drug raised due to an overdose of medicament (Afzal et al., 2022).

The dosage form that is comprised of combined gel and emulsion is referred to as an emulgel, which is formed by adding a gelling agent to either oil and water or water-in-oil emulsions. Emulgel shows diversity, as it has dual control on drug release due to the presence of both aqueous and non-aqueous phases in it (Yadav et al., 2016). Emulgels are widely employed for drug delivery across the skin. Their dermatological advantages are as follows: ease in the integration of drugs of hydrophobic nature, thixotropic, ungreased nature, ease in spreadability, ease in removal, emollient, stain-free, hydrophilicity, and greater shelf life with high and aesthetic appearance (Bhanu et al., 2011; Singla et al., 2012). In literature, a high rate of morbidity is reported due to skin and soft tissue infections (SSTIs) due to bacterial and fungal infections.

Microbes such as bacteria and yeasts often co-contribute these infections. An attractive approach for the treatment of superficial skin infections is the application of topical antimicrobials (Bhanu et al., 2011). The topical preparations show delivery of a high concentration of the drug across the skin on the targeted site with very few or no systemic side effects. However, few antibiotics, such as mupirocin and fluidic acid, show restrictions in their use due to their reported resistance (Sabri et al., 2016). Most *Escherichia coli* strains have been involved in various soft tissue skin infections that occur in patients of each age. Some infections recover without any treatment, but some require antibacterial treatments (Pagano et al., 2021). Almost 80% of the population uses medicinal plants in primary health care. When compared to synthetic drugs, herbal medicines have easy access, are inexpensive, and are safer (Afzal et al., 2022). Various natural or synthetic antimicrobials are being used to treat and delay the growth of different microorganisms. Antibiotics used in excess amount causes antibiotic resistance. Antibiotic resistance and toxicity may be developed by the use of synthetic drugs. To reduce any side effects, essential oils or natural products can be used. The parameters to detect the efficacy of effective components in the natural product include its chemical properties and concentration at active component. Flavonoids, saponins, and phenolics have antimicrobial properties. The efficacy of herbal products has gained the attention of researchers, as medicines based on plants have no side effects (Kaur et al., 2021).

*Carthamus tinctorius* L. (CT), also called safflower or false saffron, is a medicinal plant that belongs to the family Compositae or Asteraceae. This shaft-like species typically grows in dusty Southern Asia, China, India, and Iran climates. It is cultivated mainly for its seeds, which are used as edible oil and contain many useful compounds such as flavonoids, phenolic acids, fatty acids, and alkynes, which contribute to its effect as an analgesic, anti-inflammatory, antioxidant, and antimicrobial (Abdipour et al., 2019). *In silico* studies also aid in understanding the binding energies and active sites for these biological activities (Yesilyurt et al., 2020).

Therefore, the recent research aimed to develop a new topically applied emulgel that contains oil extracted from seeds of *C. tinctorius* L. plant having both antioxidant and antibacterial properties and their *in vitro* evaluation of their antibacterial activity against *E. coli* and *Staphylococcus aureus*. The antibacterial effect was compared between the formulated products available in the market and prepared emulgel. The antibacterial and antioxidant activities were checked, and the collected data were analyzed statistically.

## 2 Developed methods and materials

### 2.1 Chemicals

*C. tinctorius* oil, Carbopol 940 polymer, polyethylene glycol, methylparaben, Tween 20, Span20, *n*-hexane, methanol, propylene glycol, triethanolamine, NaOH, and potassium dihydrogen phosphate were purchased from Merck (Darmstadt, Germany), as well as distilled water, Mueller–Hinton agar (OXOID CM0337), and Nutrient broth (OXOID CM0001). All other ingredients were of analytical grade.

### 2.2 Collection, extraction, and isolation of oil from the plant

CT plants were collected from Pakistan’s tropical regions. The oil from the CT plants’ seeds was extracted using a Soxhlet apparatus (Pyrex, Germany). For extraction, *n*-hexane, a hydrocarbon having a boiling point of 68.7°C, was chosen as the preferred solvent. After extraction, oil was obtained by removing the solvent using a rotary evaporator (Javed et al., 2018a). After that, the solubility of CT oil was determined in methanol, *n*-hexane, and buffer solution (pH 5.5).

### 2.3 Design expert for experiment

The experimental permeation enhancer concentration like propylene glycol (PG) and polyethylene glycol (PEG) on emulgel was assessed using Design Expert (version 7.0.3). The quantities of PG and PEG were tested at three altitudes using a central composite rotatable design (CCRD), as indicated in **Table 1**, with other variables remaining constant throughout the study while taking into account the principal point of 0, 0 in quintuplicate.

**Table 1:** Factor arrangements: (a) concrete elements analysis with coded levels and (b) experimental design range.

Serial no. (a)	Coded level factors		PG	PEG
	X_1_	X_2_		
F1	0	0	10	3
F2	0	+1	10	4
F3	+1	0	13	3
F4	0	−1	10	2
F5	−1	0	6	3
F6	+1	+1	13	4
F7	−1	−1	6	2
F8	+1	−1	13	2
F9	−1	+1	6	4
F10	0	0	6	4
F11	0	0	6	4
F12	0	0	6	4
F13	0	0	6	4
(b).				
Coded level	−1		0	+1
X_1_ (g) PG	6		10	13
X_2_ (g) PEG	2		3	4

PG, propylene glycol; PEG, polyethylene glycol.

### 2.4 Preparation of emulgel

**Figure 1:**
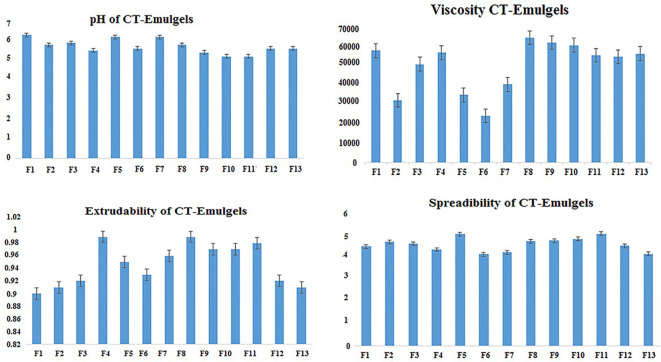
Physicochemical characteristics of CT emulgels. CT, *Carthamus tinctorius* L.

As shown in **Table 2**, 100 g of the *C. tinctorius* L. oil-based emulgel was made using various concentrations of PG and PEG. Initially, the gel phase was made with the necessary Carbopol 940 concentration to water at an appropriate speed with continuous stirring by a mechanical stirrer. Span20 was mixed with liquid paraffin, following the addition of CT oil to form the oil phase. Tween 20 was dissolved in distilled water to form an aqueous phase, and then polyethylene glycol-1000 and methylparaben were added. Then, both phases were heated to 70°C to 80°C, cooled at room temperature, and mixed to make emulsion. To develop emulgel, the emulsion was diluted with gel in a ratio of 1:1 while swirling gently (Javed et al., 2018b). **Figure 1** shows the physical appearance of the formulated emulgel.

**Table 2:** *Carthamus tinctorius* L. oil-based emulgel 100-g preparation.

Formulations	*C. tinctorius* oil % w/w	Carbopol 940 (% w/w)	X_1_ = propylene glycol (%w/w)	X_2_ = PEG (%w/w)	Tween 20 (%w/w)	Span20 (%w/w)	Methyl paraben (%w/w)	Triethanolamine	Distilled Water
F1	2.5	2	10	3	0.30	0.99	0.03	0.02	81.5
F2	2.5	2	10	4	0.30	0.99	0.03	0.02	80.15
F3	2.5	2	13	3	0.30	0.99	0.03	0.02	81.5
F4	2.5	2	10	2	0.30	0.99	0.03	0.02	82.7
F5	2.5	2	6	3	0.30	0.99	0.03	0.02	85.15
F6	2.5	2	13	4	0.30	0.99	0.03	0.02	85.15
F7	2.5	2	6	2	0.30	0.99	0.03	0.02	77.15
F8	2.5	2	13	2	0.30	0.99	0.03	0.02	79.15
F9	2.5	2	6	4	0.30	0.99	0.03	0.02	84.15
F10	2.5	2	6	4	0.30	0.99	0.03	0.02	84.15
F11	2.5	2	6	4	0.30	0.99	0.03	0.02	84.15
F12	2.5	2	6	4	0.30	0.99	0.03	0.02	84.15
F13	2.5	2	6	4	0.30	0.99	0.03	0.02	84.15

### 2.5 Physicochemical evaluation

The visual appearance, color, homogeneity, consistency, and pH of each CT-emulgel formulation were evaluated (Mottaleb et al., 2007). At 25°C, Brookfield viscometer with spindle assessed CT-emulgel formulation viscosity (Javed et al., 2018b).

#### 2.5.1 Skin irritation test

Applying CT-emulgel formulations on well-shaven rat skin, the skin irritation test was monitored for 24 h to observe unfavorable changes in morphology and color.

#### 2.5.2 Spreadability

CT-emulgel mixture with a mass of 0.1 g was placed on the transparent 1.6-cm-diameter center of a glass slide, which was then squeezed for 6 min by placing a second glass slide on it. Each CT emulgel had a centimeter-long circled spreading portion (n = 3). The following formula was used to calculate the average (Javed et al., 2018b).


(1) S=[M×L/T],

where *M* stands for the weight (g) bent to the top slide, *L* for the length (cm), and *T* for the time (seconds).

#### 2.5.3 Extrudability

The extrudability test of each CT emulgel determines if it flows freely from the collapsible tubes. The extrudability of CT emulgel was determined using a hardness tester. CT emulgel with a mass of 5 g was placed inside a collapsible tube. A pressure of 1 kg/cm^2^ was applied for 30 seconds by holding the tube with a plunger. Emulgel extruded from each tube was measured (Javed et al., 2018b).

### 2.6 Fourier transform infrared spectroscopy

By conducting Fourier transform infrared (FTIR) spectroscopy of polymers, drugs, and drug-loaded emulgels, structural factors such as polymer–drug interactions of CT-emulgel formulations were confirmed (Chakole et al., 2009). FTIR spectroscopy analyzed the values of several functional groups.

### 2.7 Stability studies and statistical analysis

The stability compartment was maintained at 40.1°C to check the stability of all CT emulgels for 6 months. After 0 months to 6 months, different physical aspects of CT emulgels, such as viscosity, pH, and drug contents, were examined (Mathias and Hussain, 2010). With Microsoft Excel 2013 statistical data, the mean and standard deviation were analyzed. Thirteen distinct formulations were compared statistically using analysis of variance (ANOVA) (Khan et al., 2020).

### 2.8 Drug release of CT emulgels (*in vitro*)

The *in vitro* drug release study of CT emulgels utilized the dissolution apparatus [USP type II]. The CT oil extract was administered as a pharmaceutical agent in phosphate-buffered saline (PBS), with a release medium at a pH of 5.5 and a temperature of 37.5°C. This was achieved by encapsulating 1 g of CT emulgel within a cellophane membrane and attaching it to the paddle of a dissolution apparatus using a thread. A volume of 500 ml of PBS was employed, while the paddle rotation speed ranged from 85 to 100 revolutions per minute (RPM). From the release media, 5 ml of the sample was removed after a predetermined amount of time and replaced with the same volume of fresh media (Shah et al., 2009). The collected sample was examined using a UV spectrophotometer (PerkinElmer, Waltham, MA, USA) to ascertain its CT oil concentration at 256 nm (Akaike, 1998). A calibration curve was applied to determine the amount of CT oil extract present in each batch of CT emulgel (Nisar Hussain Shah, 2012).

### 2.9 Thermal analysis

Thermal behavior of optimized emulgels F8 and F9 was recorded by thermogravimetric analysis (TGA) to analyze the physical state of CT oil present in emulgel formulations. The sample sizes used were 14.7 mg and 16.6 mg for F8 and F9, respectively. Under a nitrogen environment, heat stress was applied at a rate of 10°C/min from 20°C to 350°C, and thermogram peaks were recorded (Devi et al., 2021).

### 2.10 Scanning electron microscopy analysis

Scanning electron microscopy (SEM) was used to analyze the surface morphology and superficial shape of emulgels. The sampling for SEM was performed by attaching emulgels on metal slabs using adhesive tape and kept in a vacuum chamber for drying. A layer of 10-nm thickness consisting of a dense gold coating was subsequently applied using a sputter coater. Subsequently, a high-resolution SEM analysis was conducted to detect and examine the sample (Ambala and Vemula, 2015).

### 2.11 Antioxidant activity

The radical scavenging activity of 1,1-diphenyl-2-picrylhydrazyl (DPPH) was calculated to check the antioxidant activity of CT emulgels F8 and F9. At various concentrations (250 µg/ml, 500 µg/ml, 1,000 µg/ml), 1 ml from 0.1 mM of DPPH solution was prepared in ethanol and then mixed with 3 ml of the tested compounds in ethanol formulated by successive dilution. The prepared mixture was placed at room temperature of approximately 22°C and shaken for 30 min, and then the reaction mixture’s absorbance was investigated using a UV spectrophotometer at 517 nm. Different concentrations were used to detect the inhibition concentration for 50% of DPPH free radical (IC_50_). Acarbose was used as a standard antioxidant agent (Ambala and Vemula, 2015; Méndez et al., 2018). The following equation was used to calculate radical scavenging activity.

% Radical Scavenging = (1 − Absorbance of sample/Absorbance of Control) × 100.

The absorbance of control is the absorbance of pure DPPH solution.

### 2.12 Antibacterial activity

#### 2.12.1 Media preparation

Mueller–Hinton is a commonly used agar medium in microbiology for antimicrobial susceptibility testing. This agar media method was employed for the preparation of the media. To prepare the media, 38 g of agar was added to 1 L of water and then heated to dissolve it completely after autoclaving for 15 min at 121°C. This media was utilized for the agar well diffusion procedure. A nutrient broth media was created to prepare the bacterial solution by adding 13 g of powder to 1,000 ml of distilled water. After thorough mixing, this was sterilized by autoclaving for 15 min at 121°C (Ermawati et al., 2020).

#### 2.12.2 Experimental microbes

Different microbial strains, *S. aureus* (ATCC-2529) and *E. coli* (ATCC-27853) were used.

#### 2.12.3 Preparation of bacterial solution

The freshly prepared culture of *S. aureus* and *E. coli* was selected. In the next step, nutrient broth media was inoculated with bacterial colonies. To obtain precise results, nutrient media was inoculated with maximum colonies, and for each *S. aureus* and *E. coli*, an optical density greater than 1 was found.

#### 2.12.4 Plating

Petri dishes were used for plating and solidified by pouring liquefied Mueller–Hinton agar (11–14 ml). Then, the plates were labeled (Alexander et al., 2013).

#### 2.12.5 Serial dilution

To obtain a precise number of bacteria per 1 ml, serial dilutions were prepared as necessary to attain 1,000 bacteria per 100 µl. For serial dilution, there were five Falcon test tubes for each strain, and a total of 10 test tubes were used. A nutrient broth of 9 ml was added to each test tube and then labeled as 101, 102, 103, 104, and 105 for each strain of bacteria. Ten Falcon tubes were used for each strain of bacterial dilution. Nutrient broth with volume of 9 ml was added to each Falcon tube and then labeled as 101, 102, 103, 104, and 105 for each bacterial strain. From bacterial solution, 1 ml was used for Falcon tube 5, labeled as 105, and mixed and added to Falcon tube 104. The same procedure was repeated until the last one was finished, which was Falcon tube 4 (as Falcon tube 5 contained 105 per ml, same as the others). Dilution 101 with volume of 100 µl was taken via micropipette and poured on plates containing Mueller–Hinton agar, as this contained 1,000 bacteria (RaMalingam et al., 2022).

#### 2.12.6 Spreading and well formation

In the next step, 100 µl of serial dilution was selected and poured into a petri dish labeled as 4, designated for specific bacteria. Following that, with the help of swabs and loops, lawn and wells were made in petri dishes, respectively. A cotton swab was used to spread bacterial solution in multiple directions to prepare equally dispensed bacterial loon. According to the arrangement of sample per plate, 1-ml autoclaved micropipette tips were used to spread wells in the agar media.

#### 2.12.7 Sample loading and incubation

In this step, a micropipette in the range of 10 µl to 200 µl was used to pour 100 µg into the well. The sample was incubated for 24 h at 37°C. CT emulgel with a mass of 100 µg containing 2 µg of the extract was loaded in wells. The standard used was ciprofloxacin 10 μg (Wulansari et al., 2017; Chauhan, 2021). The plates were removed from the incubator after 24 h, and the diameter of the inhibition zone was measured.

### 2.13 Minimum inhibitory concentration and minimum bactericidal concentration determination

After 48-h incubation, the minimum inhibitory concentration (MIC) determined was the minimum concentration that shows no turbidity, and the minimum bactericidal concentration (MBC) determined was the minimum concentration that produces not even a single colony of bacteria. With the use of MBC and MIC, serial dilutions of oil were primed. To make serial dilutions, MIC and MBC values were prepared using the microliter broth method; with this method, a methanol extract of 10 µg/100 µl was prepared, and MIC and MBC for bacterium *E* ranges were determined using the microliter broth dilution method. This method filled the wells with 100 µl of nutrient broth to prepare serial dilutions. The ultimate oil extract concentrations in the wells were 10 µg/ml, 5 µg/ml, 2.5 µg/ml, 1.5 µg/ml, 1.25 µg/ml, 0.625 µg/ml, 0.312 µg/ml, 0.156 µg/ml, 0.078 µg/ml, and 0.039 µg/ml. After this, 100 μl of *E. coli*-based bacterial sample was added to well numbers 1–10, taken from 108 Falcon tube dilution. Then, the last two wells were marked as negative and positive controls. The negative control had only 100 µl of methanol extract solution, while the positive control had 100 µl of bacterial solution. These controls were then incubated for 24 h at 37°C. From this, MIC and MBC values were identified. In order to confirm bacterial absence, 100 µl was taken from the wells marked to have MIC and MBC values, poured into two separate nutrient agar plates, and incubated at 37°C for 18–42 h (Afzal et al., 2022).

### 2.14 Molecular docking methodology

The RCSB protein data repository included urate oxidase and *Helicobacter pylori* urease crystalline structures for virtual screening. Molecules were docked using MGLTools, AutoDock4, and Autogrid4 binary files. International Union of Pure and Applied Chemistry (IUPAC) designations were used to draw the ligand structures in ChemDraw Ultra.

The RCSB protein data repository included urate oxidase and *H. pylori* urease crystalline structures for virtual screening (Alawlaqi et al., 2023; Jabeen et al., 2023) (1R4U and 4HI0). Molecules were docked using MGLTools, AutoDock4, and Autogrid4 binary files (Morris et al., 2009). The 2D structures were depicted using the ChemDraw Ultra software (Draw, 2010), utilizing their IUPAC names. Subsequently, the Chem 3D Pro software (CambridgeSoft, 2009) was employed to perform energy minimization. The ligand structures underwent optimization and were subsequently saved in the sdf format. The Open Babel graphical user interface (GUI) software was utilized to convert these structures into the pdbqt format compatible with AutoDock. The pre-processing and visualization of proteins were conducted using the Discovery Studio Visualizer software developed by BIOVIA (Visualizer, 2005). The protein molecules in BIOVIA’s Discovery Studio Visualizer were devoid of heteroatoms, co-crystal ligands, and solvent molecules following a deletion process. After adding polar hydrogen and Kollman charges to each atom, the protein structures were saved in pdbqt format for autodocking (Ferreira et al., 2015). Structure-based virtual screening was performed using AutoDock4. The grid parameter file was created using the co-crystal ligand’s grid box dimensions (Zentgraf et al., 2007). The docking parameter files were generated using LGA and AutoDock4Zn (Morris et al., 2009). In order to ensure the reliability of the study, a total of 50 poses and 300 individuals were included in the analysis. The active sites of both proteins were subjected to docking analysis with the ligand library (Yusuf et al., 2008).

## 3 Experimental results

### 3.1 Solubility studies

*C. tinctorius* L. oil’s solubility was tested using PBS at pH 5.5 and was found to be 0.004–0.003 mg/ml. Its solubility in hexane was 0.098–0.01 mg/ml, while it was 0.092–0.01 mg/ml in methanol. These findings demonstrated the distinct solubility of *C. tinctorius* L. oil in hexane to other solvents.

### 3.2 Physicochemical characteristics

**Table 3** contains the physical characteristic analysis of the prepared *C. tinctorius* L. oil-based emulgels, including their spreadability, homogeneity, consistency, thickness, and pH.

**Table 3:** Physical characteristics of formulated CT emulgels (n = 3, mean ± SEM).

Formulations	pH	Spreadability (mean ± SEM)	Extrudability (mean ± SEM)	Viscosity (centipoise) (mean ± SEM)
F1	6.2 ± 0.033	4.420 ± 0.006	0.910 ± 0.006	53,500.000 ± 0.006
F2	5.7 ± 0.033	4.617 ± 0.003	0.923 ± 0.009	29,466.667 ± 0.003
F3	5.8 ± 0.033	4.527 ± 0.009	0.920 ± 0.006	47,000.000 ± 0.009
F4	5.6 ± 0.033	4.270 ± 0.006	0.960 ± 0.021	52,300.000 ± 0.006
F5	6.1 ± 0.033	4.970 ± 0.006	0.937 ± 0.006	31,898.000 ± 0.006
F6	5.5 ± 0.033	4.073 ± 0.003	0.927 ± 0.003	23,110.000 ± 485.215
F7	6.3 ± 0.033	4.160 ± 0.006	0.947 ± 0.009	37,236.333 ± 68.216
F8	5.7 ± 0.033	4.660 ± 0.006	0.973 ± 0.012	59,400.000 ± 152.753
F9	5.3 ± 0.033	4.677 ± 0.003	0.947 ± 0.012	57,200.000 ± 57.735
F10	5.3 ± 0.033	4.747 ± 0.003	0.950 ± 0.010	54,100.000 ± 1,154.701
F11	5.1 ± 0.033	4.977 ± 0.003	0.970 ± 0.006	51,500.000 ± 173.205
F12	5.5 ± 0.033	4.443 ± 0.007	0.947 ± 0.015	50,456.667 ± 110.504
F13	5.5 ± 0.033	4.080 ± 0.006	0.940 ± 0.015	52,233.333 ± 88.192

CT, Carthamus tinctorius L.

#### 3.2.1 Stability testing and Draize’s skin irritation test

According to the outcomes of stability testing, all CT-emulgel formulations were carried out by keeping them all for 6 months at a temperature of 40°C ± 1°C in a stability chamber. All CT-emulgel formulations had satisfactory appearances, and no significant alterations in drug content, drug release behavior, viscosity, pH, or moisture content were found. All CT-emulgel formulations underwent a 30-day skin irritation test using human volunteers. According to the findings, there were no skin lesions, irritations, or scratches. Similar findings were reported in a previously reported article (Javed et al., 2018a; Javed et al., 2018b).

#### 3.2.2 Spreadability and extrudability

According to the data presented in **Table 3** and **Figure 1**, the spreadability range of all CT emulgels prepared with Carbopol 940 as the gelling agent was observed to be approximately 4.3 ± 0.1 g·cm/s to 4.7 ± 0.1 g·cm/s. CT emulgel F9 spread easily in comparison to all other preparations (Mohite and Salunkhe, 2019). The extrudability results showed that less viscous CT emulgel had acceptable tube flow ability, while more viscous CT-emulgel preparations were difficult to extrude from tubes. The extrudability ranges were 1.272 ± 0.01, 0.961 ± 0.01, and 0.866 ± 0.01. CT emulgel F9 demonstrated extrudability of 0.961 0.01 g/cm. The results are given in **Table 3**.

### 3.3 RSM optimization result in mathematical modeling

Multiple linear regression analysis (MLRA) was employed to produce a mathematical relationship based on a polynomial equation. The positive and negative significant effects have demonstrated the comparative impacts of each factor on a given response of CT emulgels. Negative numbers represent antagonistic effects on each response, while positive values demonstrate synergistic effects.

p-Value (p 0.05) for answer response Y1 indicates linear contributions. A (PG) and B (PEG) both exhibited negligible antagonistic and cross-product effects. According to ANOVA (**Table 4**), AB (PG with PEG) demonstrated a significant antagonistic reaction, A2 (a quadratic contribution) demonstrated a significant synergistic response, and B2 demonstrated a synergistic non-significant effect. **Table 5** demonstrates multiple linear regression analysis. Here, a polynomial equation has coded factors.

**Table 4:** Analysis of multiple linear regression analysis (MLRA).

Regression coefficient	Y_1_
Model	Quadratic
Intercept	75.59
A	−0.3817
B	−1.65
AB	−4.02
A^2^	4.74
B^2^	1.08
Model (p-value)	0.01
Coefficient value %	1.79
R^2^	0.92
Adjusted R^2^	0.85
Predicted R^2^	0.74
Adjusted precision	11.60
Standard deviation	1.43
Mean	79.55
F-value	12.64

**Table 5:** Variance analysis of *Carthamus tinctorius* oil-based emulgel.

Source	Sum of square	Df	Mean square	F-value	p-Value	
Model	134.57	5	26.91	13.25	0.006	Significant
A-PG	0.98	1	0.98	0.48	0.518	Non-significant
B-PEG	18.25	1	18.25	8.98	0.0302	Non-significant
AB	65.23	1	65.23	32.12	0.0024	Significant
A^2^	50.92	1	5.92	25.07	0.0041	Significant
B^2^	2.81	1	2.81	1.38	0.2926	Non-significant
Residual	10.15	5	2.03	–	–	
Lack of fit	2.15	3	0.72	0.18	0.9023	Non-significant


(2) $${\text{Y}}_{1}=\;{\text{b}}_{0}\;+\;{\text{b}}_{1}\text{A }+\;{\text{b}}_{2}\text{B }+\;{\text{b}}_{12}\text{AB }+\;{\text{b}}_{1}^{2}{\text{A}}^{2}+\;{\text{b}}_{2}^{2}{\text{B}}^{2},$$

(3) $${\text{Y}}_{1\;=\;}75.59\;-\;0.38\text{A }-\;1.65\text{B }-\;4.02\text{AB }+\;4.74{\text{A}}^{2\;+\;}1.08{\text{B}}^{2},$$

**Figure 2:**
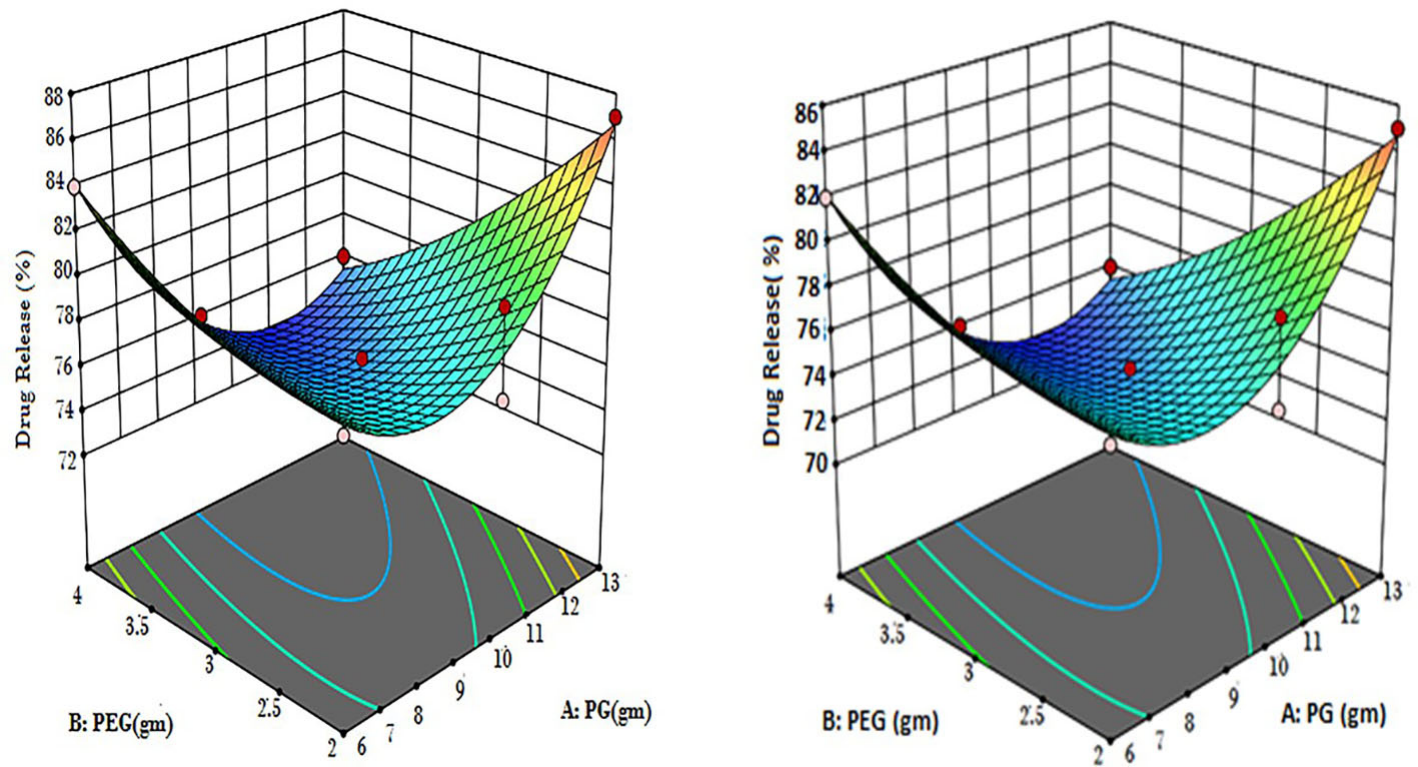
Contour 3D surface plots of optimized CT emulgels F8 and F9. CT, *Carthamus tinctorius* L.

According to the equations, it was determined that while “B” had a significant negative antagonistic impact on the release of CT oil in PBS, “A” had a relatively minor negative antagonistic effect at pH 5.5. Both of the variables (AB) demonstrated potent combined effects that demonstrated adverse (antagonistic) consequences. Propylene glycols (A2), which had a high positive (synergistic) effect, had a quadratic relationship with PEG, which is a weak positive (synergistic) relationship. Propylene glycol (A) [−0.38A + 4.74A2] showed that the percentage of drug release increased when PG concentration was higher, while the concentration of PG fell when PG concentration was lower. According to previously published research (Javed et al., 2018a), the percentage of drug release rose with higher PEG concentrations and decreased with lower PEG concentrations. This is shown by the equation PEG (B) [−1.65B + 1.08B2]. **Figure 2** displays the 3D surface plots and the contour plot. The CT-emulgel F9 formulation was chosen for further research on either humans or animals since the findings of the response surface methodology (RSM) data analysis revealed that it had the largest amount of drug release when compared to other formulations.

### 3.4 Stability testing and Draize’s skin irritation test

According to the stability findings, all CT-emulgel formulation preparations were carried out by keeping them all for 6 months at a temperature of 40°C ± 1°C in a stability chamber. The findings demonstrated that all CT emulgels have satisfactory outward appearances, and no significant alterations in drug content, drug release behavior, viscosity, pH, or moisture content were found. All CT-emulgel preparations underwent a 30-day skin irritation test using human volunteers. According to the findings, there were no skin lesions, irritations, or scratches. Similar findings were reported in previously reported articles (Hadgraft and Lane, 2016).

### 3.5 *In vitro* drug release study

**Figure 3:**
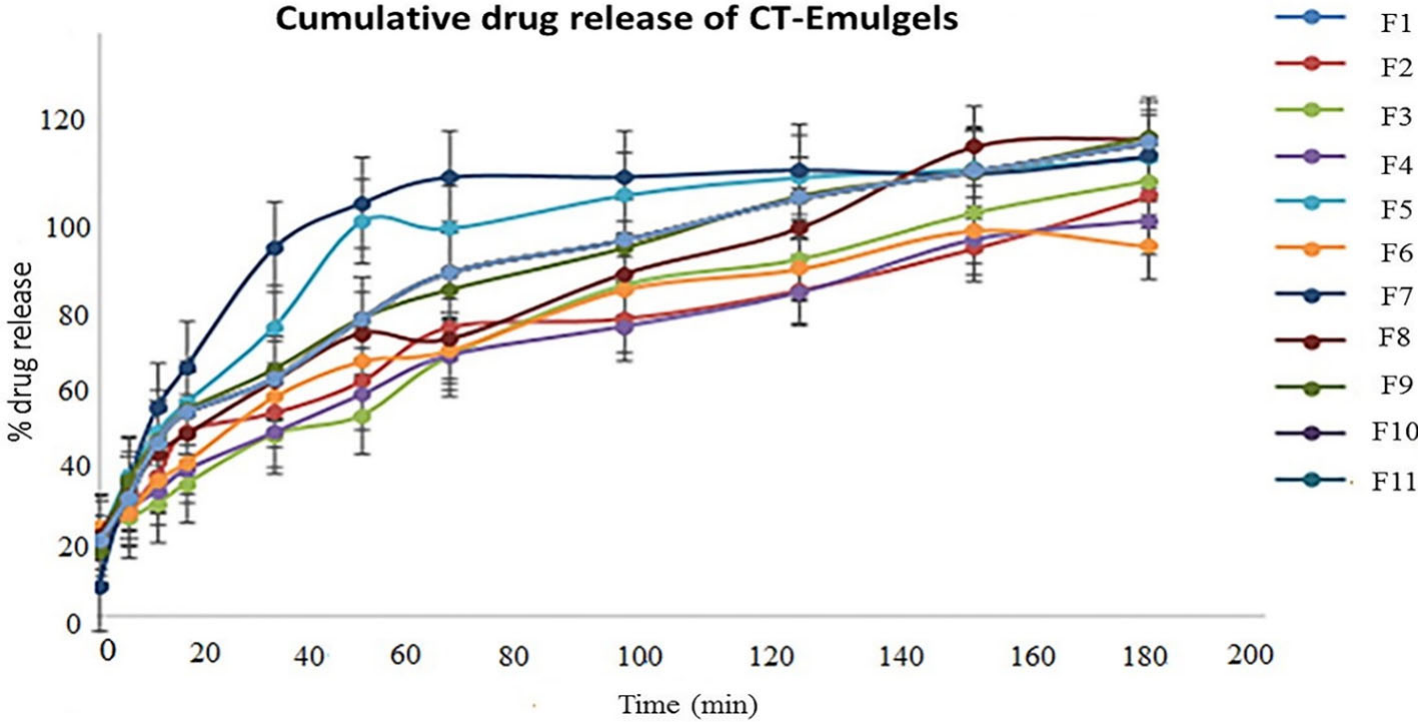
Cumulative % drug release of CT-emulgel formulations. CT, *Carthamus tinctorius* L.

*In vitro* drug release profiles of CT emulgels are shown in **Figure 3**. Drug release was observed in optimized CT-emulgel formulations in the order of F9 >F8. In comparison to other emulgel preparations, the F9 was thought to have the highest CT release (90%). Various factors, such as gelling agents, emulsifying agents, and formulation viscosity, may affect drug release. In our study, polyethylene glycol was used as a penetration enhancer, and by increasing polyethylene concentration, drug release was also increased (Burki et al., 2020).

### 3.6 Fourier transform infrared spectroscopy

**Figure 4:**
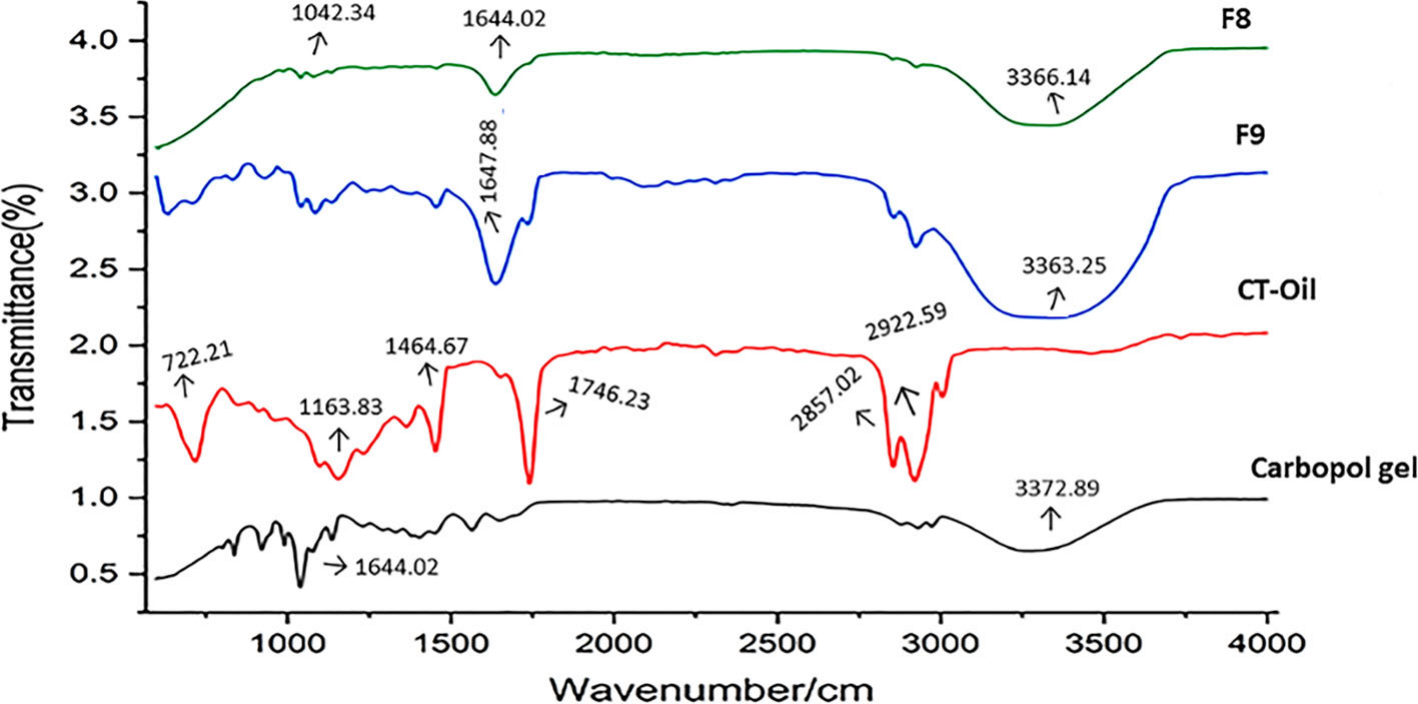
FTIR peaks of CT emulgels F8 and F9, *Carthamus tinctorius* L. oil, and Carbopol 940. FTIR, Fourier transform infrared; CT, *Carthamus tinctorius* L.

The analysis of FTIR spectra of the produced CT emulgels, *C. tinctorius* L. oil, and Carbopol 940 revealed several interesting findings, as shown in **Figure 4**. No significant differences were observed in the FTIR spectra of the CT emulgels, *C. tinctorius* L. oil, and Carbopol 940. This suggests that the formulation process and the addition of excipients did not cause major changes in the chemical composition of these components. In **Figure 4**, the amide group (–NH) in the CT emulgel exhibited broad peaks in the range of 2,800–3,800/cm. These peaks were wider than those observed in pure drug polymer. The broadening of the peaks indicates potential interactions between the amide group and other components in the emulgel formulation. The C═C alkene group showed distinct peaks in the range of 1,440–1,650/cm. According to a recent article (Javed et al., 2018a), this peak was more pronounced in the CT-emulgel formulation compared to the polymer and CT oil alone. The presence of a distinct peak in this range consistently across all formulations suggests the presence of the alkene group in the emulgel and strong bond interactions within this group. Peaks in the range of 1,120–1,180/cm were observed due to the presence of phenyl groups, while peaks in the range of 700–733/cm were attributed to the presence of hydroxyl (O–H) groups. These findings indicate the presence of specific functional groups in the CT-emulgel formulation. The stability of CT oil in all CT-emulgel formulations, including Carbopol 940, polymers, and other excipients, was determined to be satisfactory (Ayoub et al., 2015; Abdipour et al., 2019). This implies that the formulation is effective in preserving the stability of the CT oil even in the presence of other ingredients. The results are significant, as they provide valuable insights into the chemical interactions and stability of the CT-emulgel formulation. The broadening and shifting of peaks in the FTIR spectra indicate the occurrence of chemical interactions between various components in the emulgel. These findings support the suitability of the chosen formulation for the intended purpose.

### 3.7 Thermal analysis

**Figure 5:**
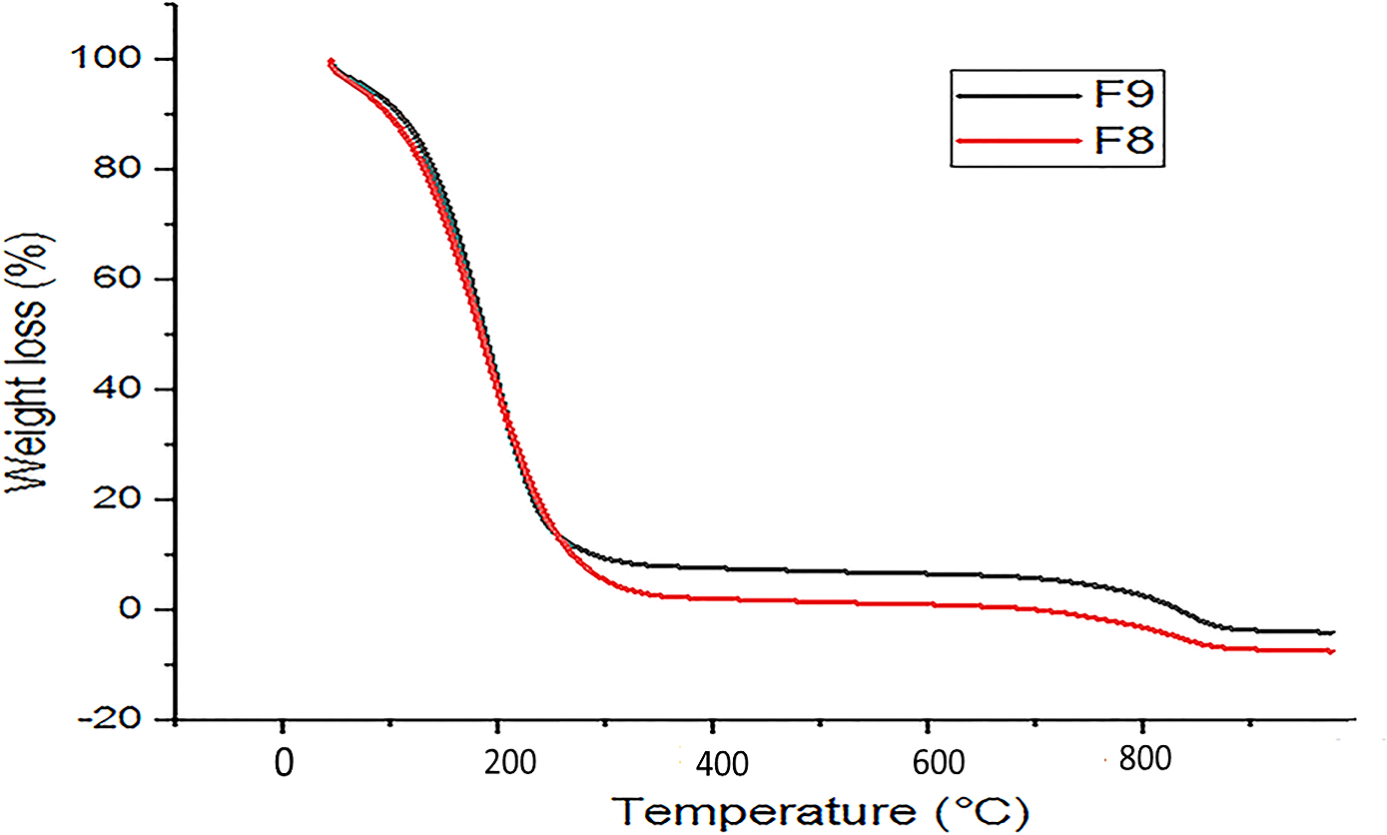
Thermal analysis of optimized CT emulgels F8 and F9. CT, *Carthamus tinctorius* L.

The TGA measurement was used to check the thermal stability of emulgel within the 25°C–350°C temperature range. The TGA plot of the emulgel is presented in **Figure 5**. The oil’s flash point is 110°C, indicating minimal degradation of the emulgel at this temperature and suggesting a stable formulation.

### 3.8 SEM analysis

**Figure 6:**
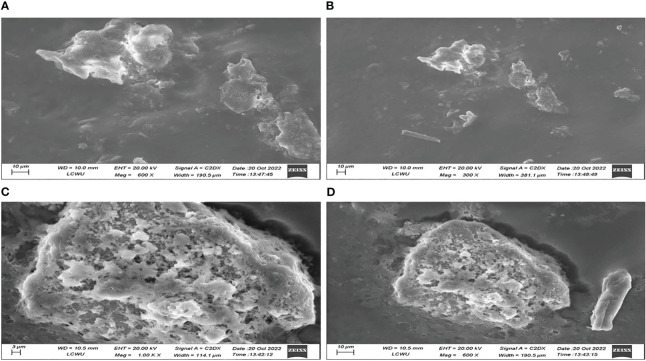
SEM investigation of CT emulgels F8 **(A, B)** and F9 **(C, D)** at different magnifications. SEM, scanning electron microscopy; CT, *Carthamus tinctorius* L.

The SEM analysis of the emulgel reveals that the surface exhibits an irregularly shaped structure, characterized by the presence of cracks and wrinkles. This observation is depicted in **Figure 6**, which illustrates samples F8 and F9. Additionally, the image reveals the presence of minute porous channels, which can be attributed to the removal of water molecules during the preparation process. The presence of various irregularly sized pockets of CT oil on the surface may be attributed to the phenomenon of polymer cross-linking within emulgel formulations.

### 3.9 Measurement of DPPH assay for antioxidant activity

The antioxidant activity of optimized CT emulgels was also checked using DPPH free radical scavenging assay. In this assay, the protons were accepted as free radicals from the antioxidant substrate, decreasing its absorbance, which was reserved as an amount of radical scavenging.

**Figure 7:**
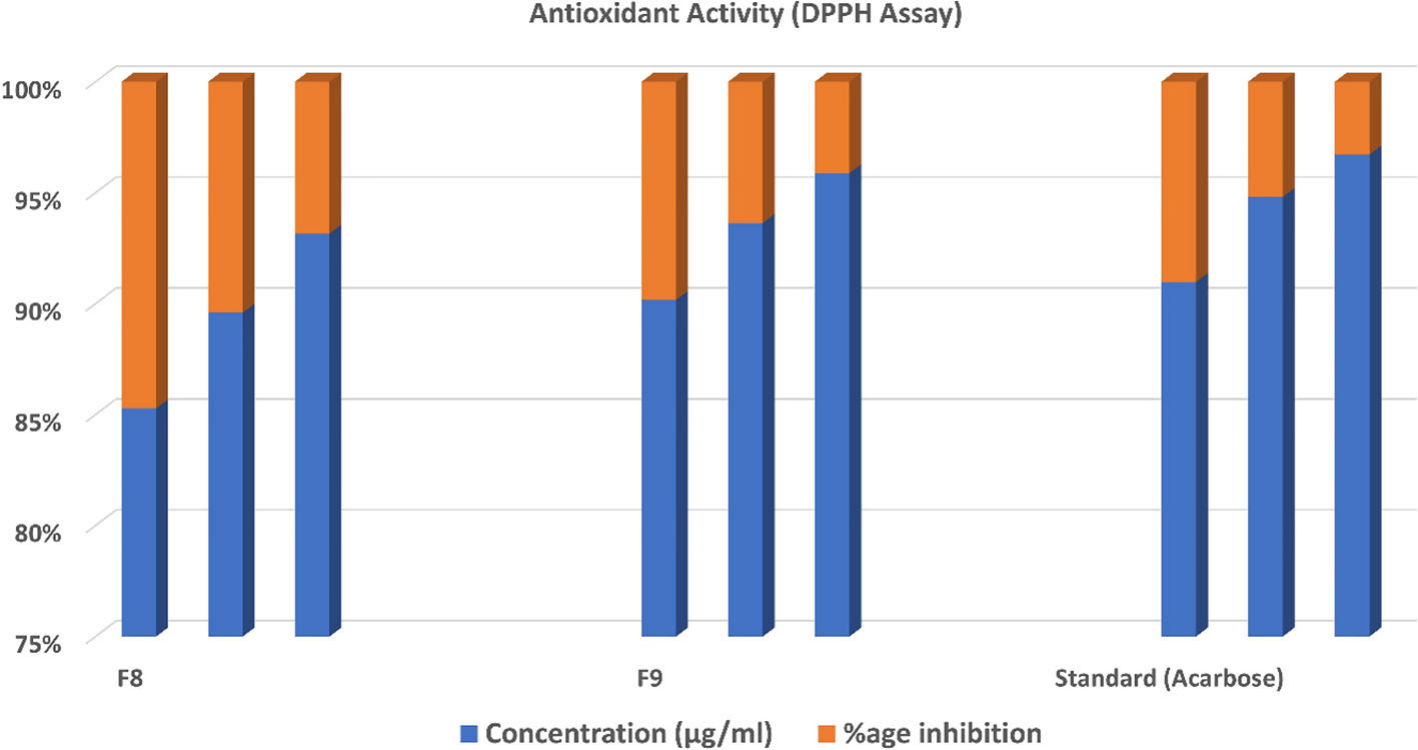
Radical scavenging activity of CT emulgels F8 and F9. CT, *Carthamus tinctorius* L.

Sample F8 had % inhibition of 42.131 ± 0.335, 56.720 ± 0.222, and 72.440 ± 0.335 at different concentrations. Sample F9 had % inhibition of 26.312 ± 0.280, 32.461 ± 0.328, and 42.762 ± 0.398 at concentrations of 250 µg/ml, 500 µg/ml, and 1,000 µg/ml, respectively. The standard drug used had % inhibition of 24.030 ± 0.578, 26.030 ± 0.619, and 33.607 ± 0.294 at concentrations of 250 µg/ml, 500 µg/ml, and 1,000 µg/ml, respectively, which shows that both samples F8 and F9 have significant antioxidant potential. The radical scavenging activity of optimized CT emulgels F8 and F9 is also shown in **Figure 7** and **Table 6**.

**Table 6:** Antioxidant activity (DPPH assay).

Sample		Concentration (µg/ml)	Absorption	% inhibition ± SEM	IC_50_ (µg/ml)
F8	1	250	1.964	42.131 ± 0.335	375.15
	2	500	1.449	56.720 ± 0.222	
	3	1,000	0.917	72.440 ± 0.335	
F9	1	250	2.5132	26.312 ± 0.280	132.8
	2	500	2.2791	32.461 ± 0.328	
	3	1,000	1.9701	42.762 ± 0.398	
Standard (acarbose)	1	250	2.598	24.030 ± 0.578	123.583
	2	500	2.509	26.030 ± 0.619	
	3	1,000	2.282	33.607 ± 0.294	

### 3.10 Antibacterial activity

**Figure 8:**
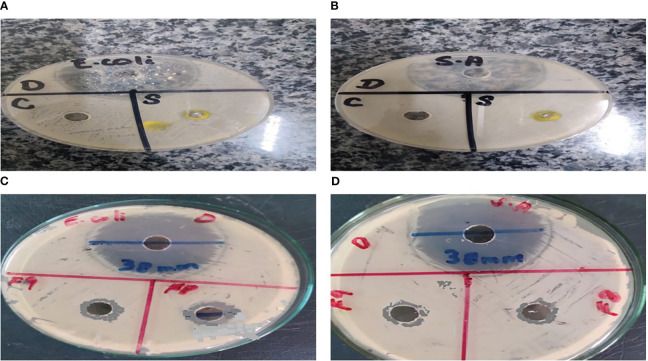
Antibacterial activity of *Carthamus tinctorius* L. (CT) oil against **(A)** *Escherichia coli* and **(B)** *Staphylococcus aureus*. **(C)** CT emulgels F8 and F9 against *E coli*. **(D)** CT emulgels F8 and F9 against *S. aureus*.

As a result of the study, it was found that *C. tinctorius* L. oil-based emulgels F8 and F9 possess antibacterial action against different test strains. The results obtained in terms of the diameter of the zone of inhibition (mm) are summarized in **Figure 8**. Bacterial strains like *E. coli* and *S. aureus* were inhibited by emulgel formulations.

To determine the antibacterial activity of various formulations of emulgel, the diameter and zone of inhibition (mm) were measured and compared. The specified amount of emulgel was taken in the recent research. It was observed that CT emulgel had significant antibacterial activity against *E. coli* and *S. aureus* as shown in **Figure 8**. The compared results of CT emulgel F8 and CT emulgel F9 indicate that samples F8 and F9 had maximum activity against *E. coli* with a small zone of inhibition toward *S. aureus*. However, pure CT oil shows a significant zone of inhibition against both bacterial strains.

A p-value of 0.00, which is less than 0.05, means that there is a significant difference existing among the means as shown in **Table 7**. The statistical analysis was conducted using the MINITAB 17 statistical package software, specifically employing the one-way ANOVA method (p< 0.05). All formulations collectively exhibited a statistically significant antibacterial effect against all tested bacteria, as indicated by a p-value of 0.00 in **Table 7**.

**Table 7:** Variance scores of CT emulgels F9 and F8 against *Escherichia coli*.

Source	DE	Adj SS	Adj MS	F-value	p-Value
CI	2	366.22	183.111	274.67	0.000
Error	6	4.00	0.66	–	–
Total	8	370.22	–	–	–

CT, Carthamus tinctorius L.

Here, F8 shows more mean area in the zone of inhibition as compared to F9, but standard drug ciprofloxacin shows a greater zone of inhibition as compared with other formulations.

**Figure 9:**
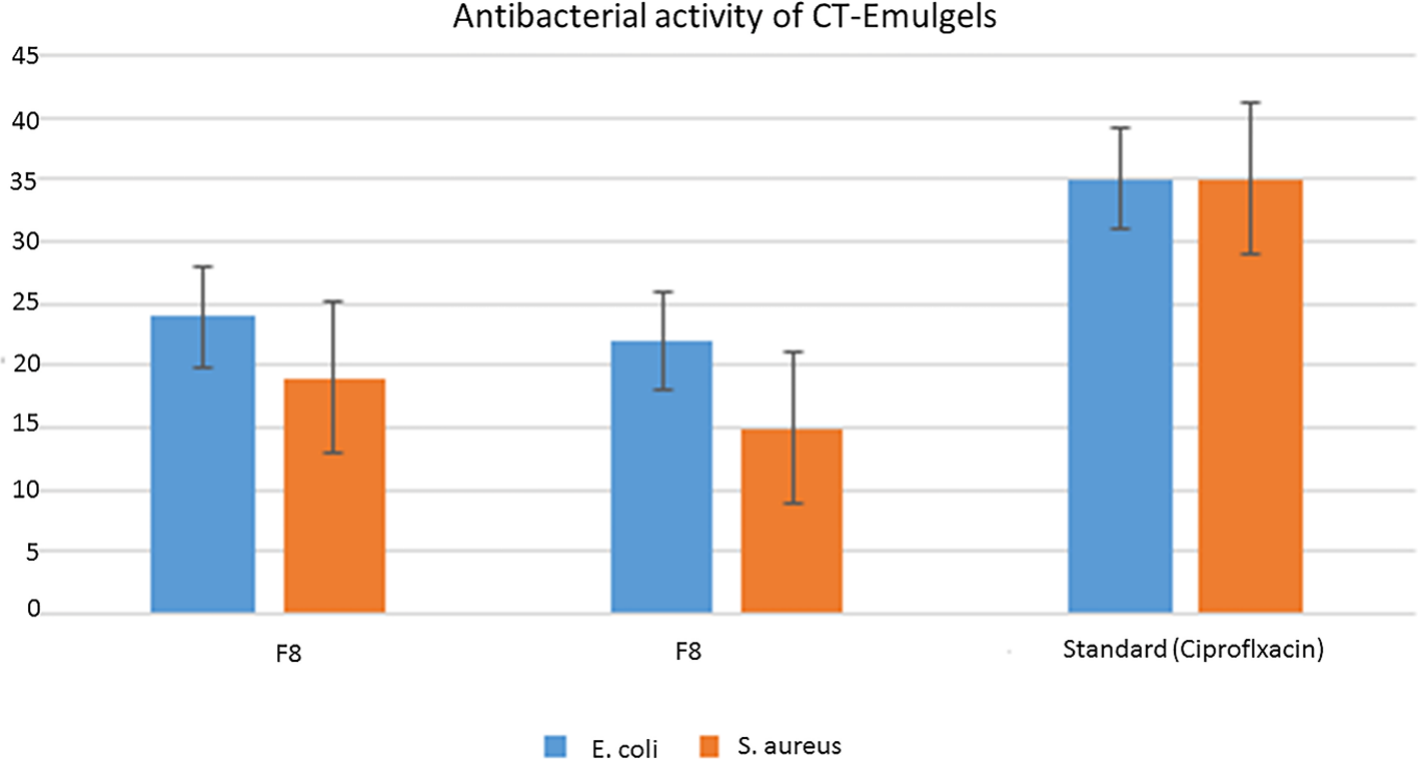
Graphical representation of zone of inhibition against bacteria *Staphylococcus aureus* and *Escherichia coli*.

Emulgel F8 shows a zone of inhibition of 24 mm for *E. coli* and 19 mm for *S. aureus*. Emulgel F9 shows a zone of inhibition of 22 mm for *E. coli* and 15 mm for *S. aureus*. Pure CT oil extract shows the zone of inhibition of 25 mm for *E. coli* and 20 mm for *S. aureus*. Ciprofloxacin used as standard shows a 36-mm zone of inhibition against both *E. coli* and *S. aureus* as shown in **Tables 8**, **9** and **Figure 9**.

**Table 8:** Mean extract zone of inhibition against *Escherichia coli* (n = 3).

C1	Bacterium	Mean	Standard deviation	95% CI
F8	*Escherichia coli*	24.333	0.577	(23.180, 25.487)
	*Staphylococcus aureus*	19.333	0.577	(18.180, 20.487)
F9	*E. coli*	21.667	0.577	(20.514, 22.079)
	*S. aureus*	15.667	1.155	(14.180, 16.487)
Standard (ciprofloxacin)	*E. coli*	36.333	1.155	(35.254, 37.079)
	*S. aureus*	35.667	0.577	(34.254, 36.079)

**Table 9:** Mean ± SEM (mm) inhibitory zone diameter.

Formulations	*Staphylococcus aureus*	*Escherichia coli*
F8	19.0 ± 0.33	24.33 ± 0.33
F9	15 ± 0.66	22.2 ± 0.66
*Carthamus tinctorius* L. oil	18.33 ± 0.33	20.60 ± 0.33
Standard (ciprofloxacin)	36.66 ± 0.66	36.66 ± 0.66

MIC and MBC values were 0.03 µg/100 µl and 0.06 µg/100 µl, respectively, for *E. coli* and 0.25 µg/100 µl and 0.05 µg/100 µl, respectively, for *S. aureus* as shown in **Table 10**. The graphical representation of the zone of inhibition against both *S. aureus* and *E. coli* is also shown in **Figure 9** and **Table 10**.

**Table 10:** *Escherichia coli* (n = 3) and *Staphylococcus aureus* (n = 3) MIC and MBC values of pure oil extract.

MIC conc. µg/100 µl Mean ± SEM	MBC conc. µg/100 µl Mean ± SEM	Bacterial strain tested
0.03 ± 0.003	0.06 ± 0.008	*E. coli*
0.025 ± 0.003	0.05 ± 0.075	*S. aureus*

MIC, minimum inhibitory concentration; MBC, minimum bactericidal concentration.

## 4 *In silico* results

The crystalline structures of urate oxidase, known for its antioxidant properties, and *H. pylori* urease, recognized for its antibacterial activity, were obtained for virtual screening. Protein Data Bank (PDB) identifiers 1R4U and 4HI0 were utilized to access these structures from the RCSB protein data library. The crystal structures of these proteins were demonstrated in their native state, with a resolution of 2.20 Å. Additionally, the crystal structures of these proteins were examined in their binding state with the investigated ligands 1_KA–10_KA. The results presented in **Tables 11**, **12** demonstrate the binding affinity, hydrogen bonding, hydrophobic interactions, and electrostatic interactions between the active site pocket amino acids and the proteins and the corresponding distances measured in angstroms.

**Table 11:** 1R4U protein ligands’ binding affinity (kcal/mol) and active site binding pocket interactions in angstrom.

Ligands with target protein 1R4U	Isolated extract compounds	Binding score in kcal/mol	Binding pocket interactions of active site
1_KA	Acacetin	−7.1	ARG176 (4.30), ARG176 (4.28), ARG176 (2.37), ASN254 (2.48), GLU259 (3.60), ARG176 (3.79), HIS256 (3.28)
2_KA	Acacetin-7-*O*-alpha-l-rhamnopyranoside	−7.4	LEU170 (4.65), TRP174 (4.57) GLU259 (3.66), ARG176 (3.69), HIS256 (3.48), TYR257 (3.22), GLU259 (3.60), THR173 (2.61)
3_KA	Acacetin-7-*O*-β-d-apiofuranosyl (1→6)-*O*-β-d-glucoside	−8.3	LEU170 (3.54), PHE258 (5.35), THR173 (2.30), GLU259 (2.20), PHE258 (5.52), LEU170 (4.79)
4_KA	3*Z*,5*E*-Trideca-1,3,5-triene-7,9,11-triyne	−4.3	ARG176 (4.92)
5_KA	3*E*,11*E*-Trideca-1,3,11-triene-5,7,9-triyne	−4.4	PHE159 (4.28), VAL227 (4.33),
6_KA	*trans*-3-Tridecene-5,7,9,11-tetrayne-1,2-diol	−4.9	ILE177 (3.00), TYR257 (2.18),
7_KA	2*Z*-Decaene-4,6-diyne-1-*O*-β-d-glucopyranoside	−5.8	ARG176 (2.37), TYR257 (2.74) ILE177 (5.06), PHE278 (4.68), ARG176 (2.33)
8_KA	8*Z*-Decaene-4,6-diyne-1-ol-1-*O*-β-d-glucuronyl-(1″,2′)-β-d-glucopyranoside	−4.4	PHE159 (4.49), ILE288 (4.92)
9_KA	8*Z*-Decaene-4,6-diyne-1-ol-1-*O*-β-d-glucuronyl-(1″,2′)-β-d-glucopyranoside	−6.6	ILE177 (2.08), HIS256 (3.19), THR173 (2.53), TYR257 (2.39), HIS256 (3.74), VAL227 (4.35), ASP175 (2.95), PHE159 (4.06)
10_KA	(2*E*)-Tetradecaene-4,6-diyne-1,10,14-triol-1-*O*-β-d-glucopyranoside	−5.8	GLU259 (2.62), GLY286 (3.59), THR173 (2.40), GLN228 (2.40), PHE159 (3.90), HIS256 (2.55)

**Table 12:** Binding affinity (kcal/mol), hydrogen binding, and hydrophobic and electrostatic interactions in angstrom for 4HI0 protein ligands.

Ligands with target protein 4HI0	Isolated extract compounds	Binding score in kcal/mol	Binding pocket interactions of active site
1_KA	Acacetin	−7.7	ALA16 (4.45) LYS146 (4.22), ASN177 (2.61) LYS146 (3.66), ARG179 (4.39), LYS146 (4.89), LYS146 (3.87)
2_KA	Acacetin-7-*O*-alpha-l-rhamnopyranoside	−8.0	GLY13 (3.74), LYS146 (3.64), LYS146 (3.87), LEU149 (4.95), ALA16 (4.07), ALA16 (4.43), ASN145 (2.55), ARG179 (2.19), ASP148 (2.53), THR15 (3.70)
3_KA	Acacetin-7-*O*-β-d-apiofuranosyl (1→6)-*O*-β-d-glucoside	−8.3	ALA16 (3.60), LYS146 (4.37), ARG179 (4.87) LYS146 (4.62), ARG179 (4.15), GLY11 (2.39), LYS14 (2.85), LYS14 (2.89), THR15 (2.61), ASP37 (2.75), VAL10 (3.28) ALA16 (3.85)
4_KA	3*Z*,5*E*-Trideca-1,3,5-triene-7,9,11-triyne	−4.4	PHE173 (4.75), PRO172 (5.05)
5_KA	3*E*,11*E*-Trideca-1,3,11-triene-5,7,9-triyne	−4.2	PHE175 (3.79), LEU157 (4.24)
6_KA	*trans*-3-Tridecene-5,7,9,11-tetrayne-1,2-diol	−4.9	ASP148 (2.43), ASN177 (2.20), ILE178 (2.45), ASN177 (1.97)
7_KA	2*Z*-Decaene-4,6-diyne-1-*O*-β-d-glucopyranoside	−6.5	LYS146 (4.12), ARG179 (4.31), THR15 (2.69)
8_KA	8*Z*-Decaene-4,6-diyne-1-ol-1-*O*-β-d-glucuronyl-(1″,2′)-β-d-glucopyranoside	−4.7	LYS165 (4.78)
9_KA	8*Z*-Decaene-4,6-diyne-1-ol-1-*O*-β-d-glucuronyl-(1″,2′)-β-d-glucopyranoside	−7.2	ASP37 (2.19), ASP37 (3.28) PHE46 (5.01), GLY11 (2.66), GLY13 (2.16), LYS14 (2.47), THR15 (2.75)
10_KA	(2*E*)-Tetradecaene-4,6-diyne-1,10,14-triol-1-*O*-β-d-glucopyranoside	−6.1	LYS146 (3.47), ARG179 (3.33), THR15 (2.62), ASP43 (2.71), ASP145 (2.04)

**Figure 10:**
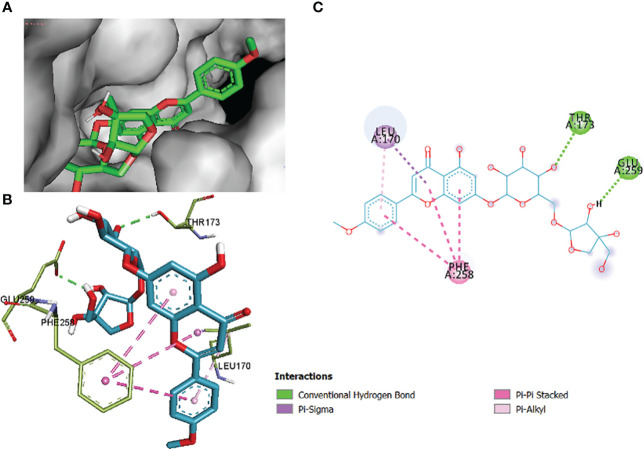
3_KA to 1R4U binding modes. Green dashes show H-bond, while other colors show hydrophobic and electrostatic interactions: **(A)** active site pocket, **(B)** amino acid interaction, and **(C)** 2D molecular interactions.

**Figure 11:**
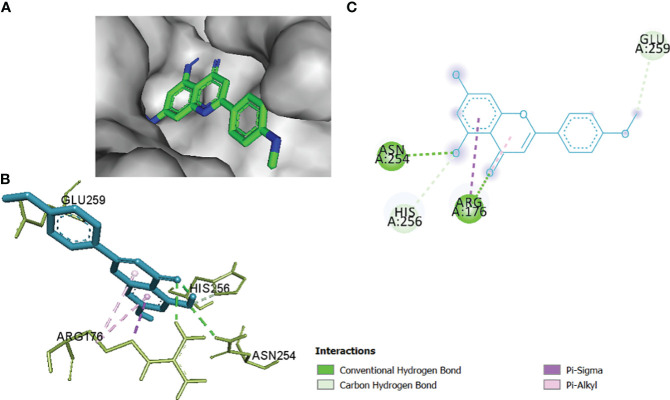
1_KA to 1R4U binding modes. Green dashes show H-bond, while other colors show hydrophobic and electrostatic interactions: **(A)** active site pocket, **(B)** amino acid interaction, and **(C)** 2D molecular interactions.

**Figure 12:**
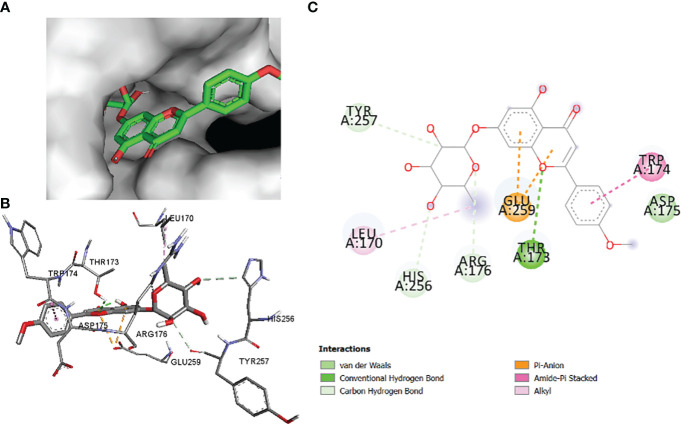
2_KA to 1R4U binding modes. Green dashes show H-bond, while other colors show hydrophobic and electrostatic interactions: **(A)** active site pocket, **(B)** amino acid interaction, and **(C)** 2D molecular interactions.

In this comparative analysis, **Table 11** shows the binding affinities and interactions of 10 ligands (1_KA to 10_KA) with protein 1R4U. The binding affinity (ΔG) is a measure of how tightly a ligand binds to a protein, with more negative values indicating stronger binding. The interactions include hydrogen bonding, hydrophobic interactions, and electrostatic interactions, which play crucial roles in stabilizing ligand–protein complexes. Among the ligands, 3_KA (**Figure 10**) shows the highest binding affinity of −8.3 kcal/mol, indicating that it forms a strong and stable complex with the protein. This ligand interacts through hydrogen bonding with THR173 and GLU259. The involvement of multiple hydrogen bonds likely contributes to its high binding affinity. Additionally, the hydrophobic interactions with LEU170 and PHE258 further stabilize the complex. In contrast, ligands 4, 5, 6, 8_KA, and 10_KA exhibit weaker binding affinities ranging from −4.3 kcal/mol to −5.8 kcal/mol. These ligands may have limited interactions with the protein, which could result in weaker binding. Notably, 4_KA has only one hydrophobic interaction with ARG176, which might explain its lower affinity when compared to others. Ligands 1_KA (**Figure 11**), 2_KA (**Figure 12**), 7_KA, and 9_KA fall in between, with binding affinities ranging from −4.4 kcal/mol to −7.4 kcal/mol. However, they exhibit multiple hydrogen bonding interactions, and their hydrophobic and electrostatic interactions appear to be less pronounced, contributing to their moderate binding affinities.

**Figure 13:**
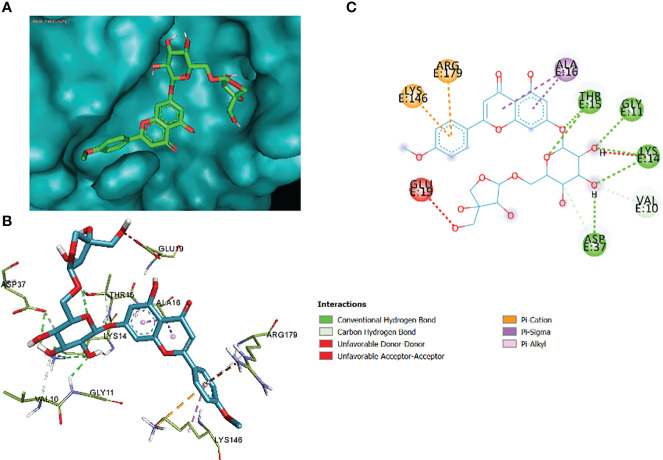
3_KA to 4H10 binding modes. Green dashes show H-bond, while other colors show hydrophobic and electrostatic interactions: **(A)** active site pocket, **(B)** amino acid interaction, and **(C)** 2D molecular interactions.

**Figure 14:**
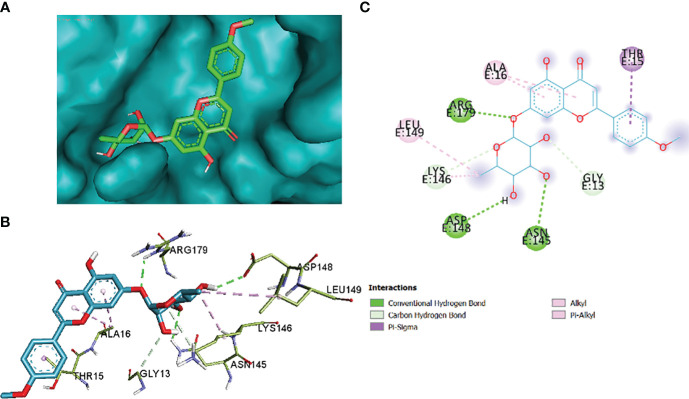
2_KA to 4H10 binding modes. Green dashes show H-bond, while other colors show hydrophobic and electrostatic interactions: **(A)** active site pocket, **(B)** amino acid interaction, and **(C)** 2D molecular interactions.

**Figure 15:**
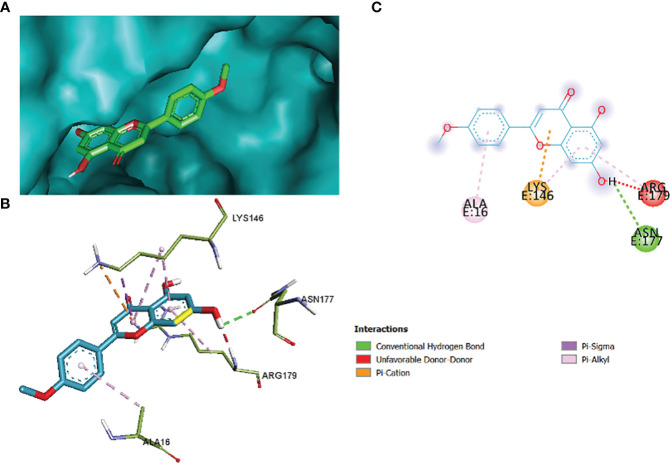
1_KA to 4H10 binding modes. Green dashes show H-bond, while other colors show hydrophobic and electrostatic interactions: **(A)** active site pocket, **(B)** amino acid interaction, and **(C)** 2D molecular interactions.

The comparative analysis of the results in **Table 12** provides the binding affinities and interactions of 10 ligands (1_KA to 10_KA) with protein 4HI0. Among the ligands, 3_KA (**Figure 13**) exhibits the highest binding affinity of −8.3 kcal/mol, indicating a strong interaction with the protein. This ligand forms hydrogen bonds with GLY11, LYS14, THR15, ASP37, and VAL10. It also engages in hydrophobic interactions with ALA16, LYS146, and ARG179. Additionally, electrostatic interactions were observed with LYS146 and ARG179. The presence of multiple hydrogen bonds, hydrophobic contacts, and electrostatic interactions likely contributes to the strong binding affinity of 3_KA. Ligand 2_KA (**Figure 14**) also demonstrates a high binding affinity of −8.0 kcal/mol. It engages in hydrogen bonding interactions with ASN145, ARG179, ASP148, GLY13, and LYS146. Hydrophobic interactions are observed with THR15, LEU149, and ALA16. This ligand also exhibits electrostatic interactions with ALA16. These interactions contribute to the favorable binding affinity observed. Ligand 1_KA (**Figure 15**) shows a binding affinity of −7.7 kcal/mol. It forms a hydrogen bond with ASN177 and engages in hydrophobic interactions with LYS146, ARG179, and ALA16. Electrostatic interactions are observed with LYS146. These interactions collectively contribute to the moderate binding affinity of 1_KA. Ligands 9_KA and 7_KA exhibit binding affinities of −7.2 and −6.5 kcal/mol, respectively. Ligand 9_KA forms hydrogen bonds with GLY11, GLY13, LYS14, THR15, and ASP37 while engaging in a hydrophobic interaction with PHE46. Ligand 7_KA forms a hydrogen bond with THR15 and interacts hydrophobically with LYS146 and ARG179. Ligands 10_KA, 8_KA, 6_KA, 4_KA, and 5_KA demonstrate lower binding affinities, ranging from −6.1 kcal/mol to −4.2 kcal/mol. These ligands may have fewer or weaker interactions with the protein. Ligand 10_KA forms hydrogen bonds with THR15, ASP43, and ASP145 while engaging in hydrophobic interactions with LYS146 and ARG179. Ligand 8_KA exhibits an interaction with LYS165, while ligand 6_KA forms hydrogen bonds with ILE178, ASN177, and ASP148. Ligands 4_KA and 5_KA show minimal interactions, with only hydrophobic contacts observed.

The comparative analysis of **Tables 11**, **12** reveals variations in binding affinities and interactions among different ligands with the respective proteins, highlighting the importance of specific molecular interactions in determining ligand–protein binding. Ligands in **Table 12** demonstrate a range of binding affinities, with 3_KA and 2_KA exhibiting the highest affinities, emphasizing the significance of hydrogen bonding, hydrophobic contacts, and electrostatic interactions in driving strong ligand–protein interactions. The comparative analysis underscores the diverse nature of ligand–protein interactions, with ligands showing varying degrees of hydrogen bonding, hydrophobic interactions, and electrostatic interactions, providing insights into the molecular mechanisms underlying ligand binding and potential implications for drug discovery and design.

## 5 Discussion

The optimized CT emulgels were evaluated for *in vitro* antibacterial activity. The disc diffusion method was used for this purpose. The diameter and zone of inhibition (mm) were measured and compared against those of bacterial strains *E. coli* and *S. aureus* as shown in **Table 10** and **Figure 8**. CT oil and optimized emulgels F8 and F9 show good antibacterial activity against both bacteria as documented in the previously published articles, which show that antibacterial activity in CT oil is due to the presence of its active components, which shows a definite mechanism that contributed to its antibacterial activity (Khémiri et al., 2020). Hiba S. et al. concluded that *Myrtus communis*, used as an essential oil, correlated to their active constituents. Wazir et al. showed that the results of antibacterial activity were also due to the occurrence of tannins and flavonoids (Ermawati et al., 2020).

The findings of a one-way ANOVA test revealed a significant value of 0.00, which indicates a probability of less than 0.05 (p 0.05). All of the tested bacteria had a p-value of 0.00, indicating that all the formulations had an antibacterial activity that was statistically significant (Ermawati et al., 2020).

CT oil showed maximum effectiveness against *E. coli* and *S. aureus*. The MIC and MBC values of pure oil were determined by serial dilution technique, which showed significant results. Similar findings have been reported by previous articles in which Wazir A. et al. documented that different methanolic extracts like *Zanthoxylum armatum*, *Terminalia bellirica*, and *Swertia chirata* show the maximum zone of inhibition against both gram-positive and gram-negative strains (Wazir et al., 2014).

According to the present research, the zone of inhibition of CT oil was 18 mm against *S. aureus* and 20 mm against *E. coli*. However, CT emulgel F8 showed a zone of inhibition of 19 mm against *S. aureus* and 24 mm against *E. coli*, and CT emulgel F9 showed a zone of inhibition of 15 mm against *S. aureus* and 22 mm against *E. coli*.

The antioxidant activity of optimized CT emulgels was also checked using DPPH free radical scavenging assay. In this assay, the protons were accepted as free radicals from the antioxidant substrate, decreasing its absorbance, which was reserved as an amount of radical scavenging. Therefore, antioxidant activity was measured by DPPH assay as previously reported by Wazir et al. (2014).

Sample F8 had % inhibition of 42.131 ± 0.335 to 72.440 ± 0.335 at different concentrations. Sample F9 had % inhibition of 26.312 ± 0.280 to 42.762 ± 0.398 at concentrations in which both samples F8 and F9 have significant antioxidant potential.

The CT oil was richer in flavonoids and phenolic acids, and antibacterial activity was due to the presence of these components as documented in a previously reported article (Kozłowska et al., 2022).

The previously reported articles concluded that the presence of endogenous free radicals causes oxidative damage, which was protected by the presence of flavonoids and phenolic acids (Chandra and Saklani, 2017; Kozłowska et al., 2022). Kozowska M. et al. also reported that the results of antibacterial and antioxidant activities were also correlated with a high amount of phenolic acid (Kozłowska et al., 2022).

## 6 Conclusion

This study successfully developed optimized herbal emulgel formulations (F8 and F9) containing *C. tinctorius* L. oil. These formulations exhibited excellent physicochemical properties. This research provided compelling evidence of these medicinal plants’ antimicrobial and antioxidant properties without any reported side effects. In addition, the emulgel was effective against *E. coli* and *S. aureus in vitro*, making it a potential treatment for bacterial skin infections. The emulgel was also biocompatible with human skin, which is a good sign of its safety for topical use. This study presents a new, cost-effective topical emulgel formulation containing *C. tinctorius* L. oil as a potential alternative for treating bacterial skin infections. The results cover the methods for additional research to fully understand the mechanisms of action of emulgels in treating bacterial infections.

Furthermore, the study conducted a comparative analysis of molecular docking with antibacterial and antioxidant protein targets. The *in silico* results highlighted the diverse interactions between ligands and proteins, such as hydrogen bonding, hydrophobic interactions, and electrostatic interactions. These findings offer insights into the molecular mechanisms underlying ligand binding, which can have implications for future drug discovery and design. This study will focus on identifying and isolating the pure compounds from the emulgel formulations. This will help determine the individual biological effects of these compounds as antibacterial and antioxidant agents. By doing so, the study aims to contribute to developing new and safer treatment options for bacterial infections and conditions related to oxidative stress. The findings have implications for future drug development and highlight the importance of exploring the therapeutic benefits of medicinal plants in a quest for novel treatments with reduced side effects.

## Data availability statement

The raw data supporting the conclusions of this article will be made available by the authors, without undue reservation.

## Ethics statement

The current research is approved by the ethical committee of the Faculty of Pharmacy, Bahauddin Zakariya University, under 196/PEC/2022. Informed consent was obtained, and ethical guidelines were followed. The approval letter confirms that all necessary precautions were taken to ensure the protection of human subjects and adherence to ethical standards. Notably, all animal experimentations were conducted in accordance with applicable laws, regulations, and guidelines, prioritizing animal welfare and minimizing any potential harm.

## Author contributions

JS and SS conceptualized the study, performed the experiments, and wrote the manuscript. HJ performed review and editing. AsA, AnA, and TZ performed a formal analysis and refined and improved the manuscript. SS supervised, edited, and conceptualized the study. FS supervised the study, performed *in silico* work, and contributed to manuscript refinement and improvement. GW, YJ, H-AN, and MB performed project administration, review and editing, and data validation. All authors contributed to the article and approved the submitted version.
